# Adherence to ivermectin is more associated with perceptions of community directed treatment with ivermectin organization than with onchocerciasis beliefs

**DOI:** 10.1371/journal.pntd.0005849

**Published:** 2017-08-14

**Authors:** Fanny Nadia Dissak-Delon, Guy-Roger Kamga, Perrine Claire Humblet, Annie Robert, Jacob Souopgui, Joseph Kamgno, Marie José Essi, Stephen Mbigha Ghogomu, Isabelle Godin

**Affiliations:** 1 Ministry of Public Health, Yaoundé, Cameroon; 2 Université Libre de Bruxelles, Brussels, Belgium; 3 University of Buea, Buea, Cameroon; 4 Epidemiology and Biostatistics Research Division, Institut de recherche expérimentale et clinique, Université catholique de Louvain, Brussels’ campus, Brussels, Belgium; 5 Centre for Research on Filariasis and other Tropical Diseases, Yaoundé, Cameroon; 6 University of Yaoundé I, Yaoundé, Cameroon; Institute of Medical Microbiology, Immunology and Parasitology, GERMANY

## Abstract

**Background:**

The fight against onchocerciasis in Africa has boomed thanks to the Community Directed Treatment with Ivermectin (CDTI) program. However, in Cameroon, after more than 15 years of mass treatment, onchocerciasis prevalence is still above the non-transmission threshold. This study aimed to explore a possible association between people’s beliefs/perceptions of onchocerciasis and of CDTI program, and their adherence to ivermectin in three regions of Cameroon.

**Methodology/Principal findings:**

A cross sectional survey was carried out in three health districts with persistent high onchocerciasis prevalence. Participants were randomly selected in 30 clusters per district. Adherence to ivermectin was comparable between Bafang and Bafia (55.0% and 48.8%, respectively, p>0.05) and lower in Yabassi (40.7%). Among all factors related to program perceptions and disease representations that were studied, perceptions of the program are the ones that were most determinant in adherence to ivermectin. People who had a “not positive” opinion of ivermectin distribution campaigns were less compliant than those who had a positive opinion about the campaigns (40% vs 55% in Bafang, and 48% vs 62% in Bafia, p<0.01), as well as those who had a negative appreciation of community drug distributors’ commitment (22% vs 53% in Bafang, 33% vs 59% in Bafia, 27% vs 47% in Yabassi; p<0.01). The most common misconception about onchocerciasis transmission was the lack of hygiene, especially in Bafia and Yabassi. In Bafang, high proportions of people believed that onchocerciasis was due to high consumption of sugar (31% vs less than 5% in Bafia and Yabassi, p<0.001).

**Conclusion/Significance:**

There are still frequent misconceptions about onchocerciasis transmission in Cameroon. Perceptions of ivermectin distribution campaigns are more strongly associated to adherence. In addition to education/sensitisation on onchocerciasis during the implementation of the CDTI program, local health authorities should strive to better involve communities and more encourage community distributors’ work.

## Author summary

Onchocerciasis control in Africa has boomed with the ‘Community Directed Treatment with Ivermectin’ program. Successes of this program over many African countries prompted the international community to set it as main strategy for elimination of onchocerciasis and lymphatic filariasis. In Cameroon, onchocerciasis prevalence in some areas is still higher than what was predicted, despite more than 15 years of mass treatment. Therefore, in the frame of enlargement of the treatment zones, there is a need for enriching local literature on changeable factors that influence adherence to ivermectin. In this study, we assessed possible association between systematic ivermectin intake and people’s beliefs/perceptions of the disease and the program in three neighbouring regions of the country. Beliefs on onchocerciasis and their associations with adherence may differ from one community to another within the same area of the country. Furthermore, among all the factors related to disease and program representations that were studied, perceptions of ivermectin campaign’s organization and of community drug distributor’s commitment are the ones that were most determinant in adherence to ivermectin. Efforts should be done by health authorities in the program implementation inside the communities, with better population ownership and ensuring of good community drug distributors’ work.

## Introduction

Onchocerciasis, also known as *river blindness* is a vector-borne parasitic disease caused by a very thin worm, *Onchocerca volvulus*, and transmitted by the bites of black flies belonging to the *Simulium* species. The burden of the disease is observed more in Africa, especially among the poorest population of the continent [1]. In fact, onchocerciasis is responsible for debilitating eyes and skin lesions, having negative psychosocial and economic impact at local (communities) and even national levels in the countries affected [2,3].

Since 1995, onchocerciasis control activities focus on the Community Directed Treatment with Ivermectin (CDTI), a strategy adopted in African countries by the African Program for Onchocerciasis Control (APOC) consisting of yearly mass administration of ivermectin. Multiple studies show evidence of remarkable improvement in the ivermectin treatment coverage with the CDTI strategy, thus contributing to successful reductions in onchocerciasis transmission and prevalence [3–6]. However, ivermectin is only effective on Onchocerca microfilariae and has no known lethal effect on adult worms, which reproductive lifespan is estimated to about 14 years [7]. This is one of the main reason why, to achieve the onchocerciasis elimination goal, ivermectin treatment must be repeated annually for at least 15 years without reinfection [3]. Considering this, onchocerciasis corresponds to the “*long period of supervision*, *observation and care*” criterion to be classified as a chronic disease, with regards to the definition adopted by WHO in 2003 [8]. As with many chronic diseases, the major issue of onchocerciasis control and elimination is adherence to mass drug administrations (MDA).

The WHO adherence project in 2003 defined adherence to be, the “*extent to which a person’s behaviour–taking medication*, *following a diet*, *and/or executing lifestyle changes*, *corresponds with agreed recommendations*” [8]. One of the critical factors taken in account during the elaboration of the conceptual framework of onchocerciasis elimination process was the treatment coverage [3], which is the percentage of population taking the drug in a given area during a given MDA. However, treatment coverage gives a picture at community level, whereas adherence considers each individual inside the community. In other words good coverage does not necessarily guarantee good adherence (the same individuals take the drug each year), as shown by Brieger *et al*., in communities with optimal coverage they found ‘*a fairly large pool of people who had taken in less than three times in the 8 years since the programme began*’ [9]. Evidence from several studies shows that the individuals who don’t take ivermectin each year may provide sources of reinfection for their communities [9–12].

The challenge of adherence in the specific context of onchocerciasis is enhanced by the fact that in the frame of MDA, all inhabitants in a selected zone, even if those who are apparently in good health, have to take the drug. For instance in a zone of 50% of onchocerciasis prevalence, there are 50% of “healthy” people (but with high risk of developing the disease) who have to comply in a yearly treatment which is accompanied by constraints such as stop alcohol intake the day of treatment, and potential side effects including tiredness with resultant impact in their daily activities, knowing that alcohol consumption and fear of side effects are documented reasons for ivermectin avoidance [13,14]. More generally, the main factors associated with compliance to ivermectin found in the literature include non-modifiable characteristics like age and gender, and also modifiable factors such as perceived susceptibility to onchocerciasis, perception of program organization, social influence and support, and perceived benefits and risks of ivermectin [10,12,15–17].

In addition to those studies, it is really valuable to have further documentation about the importance and the balance of the main factors related to compliance, especially in the frame of the new resolutions for onchocerciasis elimination within the 2016–2025 period which include intensification of MDA and extension of pre-existing CDTI zones [18].

Moreover, it is also important to have further documentation on adherence in the context of a country like Cameroon, where despite 15 years of treatment onchocerciasis prevalence is persistently high in many CDTI zones, remaining above the expected prevalence as predicted in the elimination process [19,20].

The aim of this study was therefore to explore possible associations between people’s perceptions of CDTI program, their beliefs on onchocerciasis, with their adherence to ivermectin in three Cameroonian Health Districts (HDs), located in three different regions of the country where onchocerciasis prevalence is still above the transmission threshold.

## Methods

### Study design

We conducted a cross sectional cluster study, with 30 clusters in each health district corresponding to 30 different communities. These communities were randomly selected using a proportional-to-population size method based on sizes from the 2014 CDD census in the targeted HDs. Data collection was done using a pre tested questionnaire administered to participants by trained research assistants.

### Study sites

The study was done between April and June 2015 in three HDs implementing the CDTI program in Cameroon: Bafang, Bafia and Yabassi. Located in 3 border regions in the Centre-West area of Cameroon, those HDs have different prevalence of onchocerciasis. They have in common agriculture as main productive activity, and French as predominant official language of communication.

#### Bafang Health District

Located in the West Region at about 300 km north of Yaoundé, the capital city of Cameroon, the Bafang HD is part of the “West” CDTI Project in Cameroon, where MDA started in 1996. In 2014, the Community Drug Distributors (CDD) counted 82,346 inhabitants. The hydrography made of many streams all over the district (with their seasonal tributaries), that originate in the mountains and flow into the Nkam River, provide favourable breeding sites for Simulium sp. flies.

#### Bafia Health District

The Bafia HD belongs to the “Centre 1” CDTI Project in Cameroon, which was launched in the year 1999 after 4 years of ivermectin clinical trial and mass treatment in some communities of the District. The Bafia HD is located in the Centre Region, at 120 km from Yaoundé. According to the 2014 CDD census, the population of this HD is estimated at 226,117 inhabitants. It is a forest-savanna transition zone, irrigated by many fast flowing rivers like Sanaga and its tributaries, the Mbam and the Noun streams constituting ideal breeding sites for Simulium sp.

#### Yabassi Health District

Located in the Littoral Region, this District is included in the “Littoral” CDTI Project in Cameroon, which was launched in the year 1999. The Yabassi HD is 100 km from Douala, the economic capital of Cameroon. According to the 2014 CDD census, the population of this health district is estimated at 21,459 inhabitants. This HD is irrigated by many fast flowing rivers like Nkam, Dibamba, Mabombé, Njanga and Mahé which are favourable conditions for blackfly breeding.

The HDs were selected according to the evolution of their onchocerciasis prevalence from baseline studies carried out between 1991 and 1999 to the follow-up surveys in 2011 and lastly a recent survey conducted in 2015, one month before the beginning of our data collection. In order to ensure variability, we chose 3 HDs in different regions of Cameroon, with also different levels of onchocerciasis endemicity.

Bafang HD had a significant decrease in onchocerciasis prevalence: in the communities of Bakassa and Bakonti the percentage of positive microfilaria dropped from 59.0% and 69.2% in 1996, to 5.6% and 3.6% in 2011, respectively [19]. In the other hand, the decrease in microfilaria prevalence in Bafia and Yabassi HDs was less pronounced.

In Bafia HD, onchocerciasis prevalence in 1991 was up to 91% among men, and 54% among women in the Biatsota community [21]. In 2011, a survey conducted in the *Centre 1* CDTI zone where Bafia HD is located showed a prevalence of 53.5%, with a maximum village prevalence of 71.6% [20]. Kamga *et al*. in 2015 found a microfilaria prevalence of 45.8% in the community of Biatsota [22].

Finally, the 2011 survey showed a prevalence of 48.2% (maximum village prevalence: 69.4%) in the *Littoral 2* CDTI area where Yabassi HD is found [20], while in 2015 onchocerciasis prevalence was estimated at 12.3% in the community of Bonadissake, Yabassi HD [22].

### Participant sampling

Sample size computation in each HD was based on the hypothesis that at least 50% (non-informative prior prevalence) of respondents will have a positive perception on onchocerciasis and CDTI program, using a 10% margin error, a design effect of 4 for correcting for a high intra clusters correlation, and assuming a non-respondent loss rate of 10%. Under such assumptions, the minimal size for each HD was 421 subjects.

To obtain those 421 individuals in each HD, we selected 30 clusters, corresponding to 30 different communities. These communities were randomly selected using a proportional-to-population size method based on sizes from the 2014 CDD census in the targeted HDs. Since it is quite difficult to get an accurate list of households, especially in the rural areas of Cameroon, the households in each community were randomly selected by the research assistant who took the direction indicated by the bottle neck after turning a bottle on the ground, from a central point within the community (the health centre of the village or the chief’s house) and taking all households located in his right side until reaching the required number of households (421 households to have 421 respondents). In the event that he found an endpoint, he returned to the main point of the village and repeated the bottle operation. In each household, the respondent was selected among the present eligible members by tossing a coin, after the approval of the head of household. In case the head of household rejected the toss, he/she was given the opportunity to select the respondent of his choice. All individuals found in the households were eligible for the interview except: visitors, people aged less than 15 years and those who couldn’t understand nor answer in French.

### Data collection and analysis

Data were collected by 10 trained research assistants (RAs), each of them were responsible for the administration of 43 face-to-face interviews per district. The collection instrument consisted of a French-structured questionnaire set up to provide socio-demographic information: age, sex, level of instruction, occupation, marital status, religion, ethnicity, length of stay in the village. The other information of the questionnaire concerned people’s beliefs on onchocerciasis, their perceptions of CDTI program, and information on adherence to ivermectin. Some of these questions included experience/opinion on onchocerciasis transmission, severity and curability; opinion on ivermectin efficacy; appreciation of ivermectin MDA campaigns organization and usefulness; and opinion on CDDs work. Prior to data collection, a pre-test of the questionnaire was done in each HD, in the villages/communities far from those selected for the study. During the pre-test, it was realized that most of the respondents were neither familiar with the name/acronym “onchocerciasis” nor with “CDTI Program”. These name/acronyms were thus replaced respectively by “filaria” and “*Mectizan Campaign*”.

Respondents’ beliefs on onchocerciasis (filaria) were assessed by seeking their opinions on transmission, filaria severity and possible treatment, using multiple choices semi-closed questions. Their perceptions of CDTI Program was obtained by asking semi-closed questions on opinions about ivermectin MDA Campaigns, CDDs commitment and ivermectin efficacy.

Adherence to ivermectin was defined by systematic (regular) intake during each campaigns, assessed by asking to the participants if they take ivermectin every year since they started taking it. This question was asked after two previous “put in track” questions concerning ivermectin intake at least once in their life, and ivermectin intake during the last campaign.

Data were analysed with the statistical software Stata version 13. Proportions were compared using Chi^2^ tests in univariate analyses. Variables associated with adherence to treatment in univariate analyses were then submitted to a multiple logistic regression analysis and associations were expressed as adjusted odds ratios (AOR) with 95%confidence interval. A p-value lower than 0.05 was considered as significant.

### Ethical considerations

Before beginning the survey, ethical approvals were obtained from both the National Ethics Committee for Human Health Research (N°2015/01/543/CE/CNRESH/SP) in Cameroon, and the Institutional Review Board of the Université catholique de Louvain, Brussels campus in Belgium. Additionally, a Research Administrative Authorization was granted by the Cameroon Ministry of Public Health (N°631–1315).

We also received approvals (verbal or written) from the Permanent Secretary of the National Onchocerciasis Control Program, the Regional Delegates of Public Health for the West, Centre and Littoral Regions, and the District Medical Officers of Bafang, Bafia and Yabassi. The chiefs of villages/communities were also met at the arrival of RA.

Prior to each interview in the household, the RA obtained written consent of the household head or his representative as well as from the final respondent in the household. The chief of household was given the opportunity to choose the respondent, and the respondent was informed about his right to end the interview at any time.

## Results

### Study population

Across the 3 HDs 1,378 households were identified, amongst which 89 (6.5%) were excluded because of participation refusal. The main reasons of refusal were: busy schedule (going to farm, cooking), tiredness (coming from farm/work), or lack of interest on the study. Only 17 households (1.2%) were excluded because of local language barriers.

Among the 1,378 households 1,272 (92.3%) were visited. One member per household was interviewed, making a final sample size of 1,272 individuals: 430 in Bafang, 421 in Bafia, and 421 in Yabassi. The median age (IQR) was 35 (24–50) years in Bafang, 40 (27–52) years in Bafia, and 37 (27–52) in Yabassi. Women were predominant in Bafang (57.4%) but not in Bafia nor in Yabassi (46.8% and 48.7%, respectively); the other socio economical characteristics such as education, employment and length of stay in the villages are described in Table 1.

**Table 1:** Sociodemographic characteristics of the respondents in Bafang, Bafia and Yabassi health districts.

	Health district		
	Bafang (n = 430)	Bafia (n = 421)	Yabassi (n = 421)
**Age, years**			
*Median (IQR)*	35 (24–50)	40 (27–52)	37 (27–52)
*Range*	15–82	15–86	15–90
**Sex, n (%)**			
*Female*	247 (57.4)	197 (46.8)	205 (48.7)
**Level of instruction, n (%)**			
*No formal*	28 (6.5)	35 (8.3)	32 (7.6)
*Primary*	92 (21.4)	164 (39.0)	131 (31.1)
*Secondary 1*	137 (31.9)	122 (29.0)	125 (29.7)
*Secondary 2 and above*	173 (40.23)	100 (23.75)	133 (31.6)
**Principal occupation, n (%)**			
*No occupation*	207 (48.1)	161 (38.2)	182 (43.2)
*Farmer/trader*	171 (39.8)	216 (51.3)	180 (42.8)
*Employed*	52 (12.1)	44 (10.5)	59 (14.0)
**Length of stay in the village, years**			
*Median (IQR)*	14 (6–26)	20 (9–39)	13 (4–31)
*Range*	0–82	0–80	0–80

### Onchocerciasis recognition and definition

The term “onchocerciasis” was not well known among the respondents in the 3 HDs: “onchocerciasis” was familiar to 47.0% of them in Bafang, 63.0% in Bafia and 66.3% in Yabassi (p<0.001). Moreover, more than one third of the respondents who knew the word “onchocerciasis” was unable to give/remember its definition (48.5% in Bafang, 32.5% in Bafia and 36.2% in Yabassi).

Three principal ways of defining onchocerciasis arose from respondents spontaneous answers. Firstly, people defined onchocerciasis by referring to its cause: for instance, disease caused by insects/mosquitoes/black fly bites or by dirt/dirty water. Secondly, onchocerciasis was defined by referring to its consequences: disease which causes skin/eye problems for instance, and thirdly, onchocerciasis was defined by its synonym: onchocerciasis is river blindness for example.

“Filaria” or “filariasis” was cited by some respondents, either as a synonym or as a consequence of onchocerciasis.

### Beliefs on onchocerciasis and CDTI perceptions

#### Beliefs on onchocerciasis

**Fig 1:**
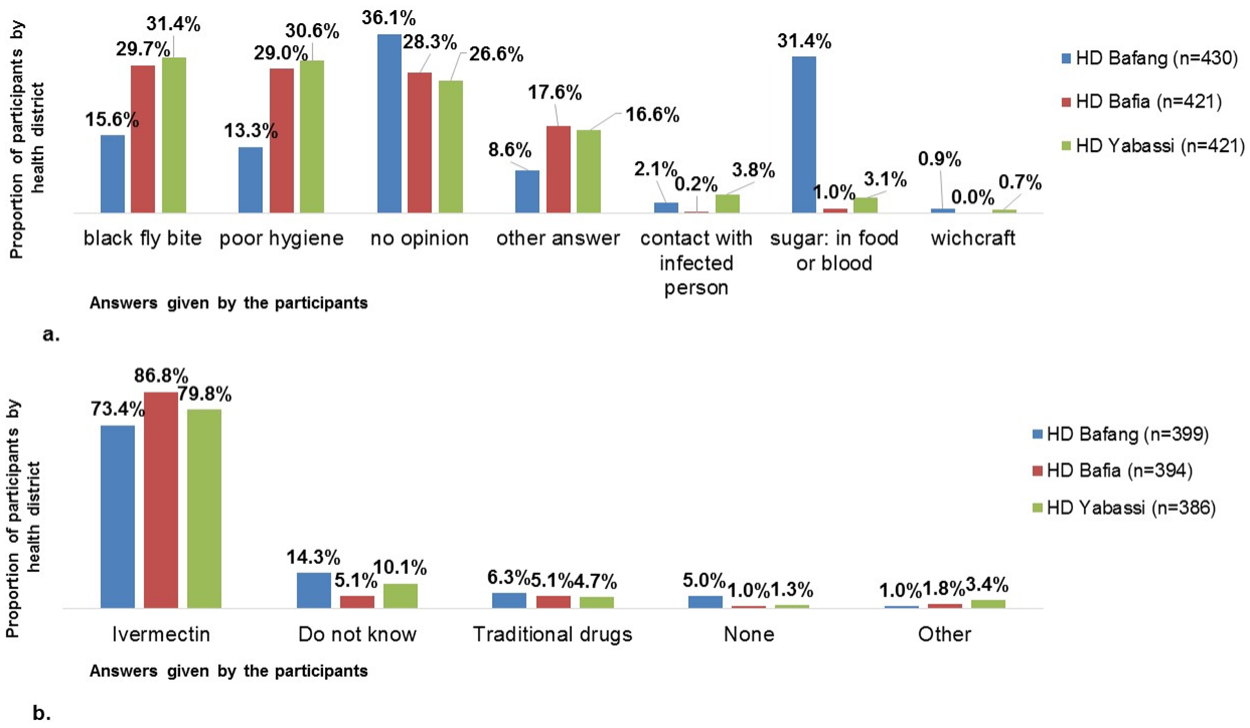
Beliefs on onchocerciasis in Bafang, Bafia and Yabassi health districts. (a) Beliefs on filaria causes*. (b) Opinion on best treatment for filaria** *: Multiple answers possible. **: Only for those who said that filaria is curable.

To the question about identification of onchocerciasis (filaria) causes, only 15.6% cited black fly bite in Bafang HD, as against 29.7% and 31.4% in Bafia and Yabassi respectively, p<0.001 (Fig 1a). Higher proportion of people believing that filaria was caused by a high sugar consumption or high blood sugar levels were also found in Bafia (31.4%, versus 1.0% and 3.1% in Bafia and Yabassi respectively, p<0.001, Fig 1a). The most common misconception about onchocerciasis transmission was related to poor hygiene conditions (Fig 1a), namely in Bafia and Yabassi where respectively 29.0% and 30.6% shared that misconception as against 13.3% in Bafang HD (p<0.001, Table 2).

**Table 2:** Comparison of beliefs on onchocerciasis and CDTI perceptions between Bafang, Bafia and Yabassi health districts.

Beliefs on onchocerciasis	Health District	Answer given	Chi-square test p value
		Yes (n, %)	
**Perceived transmission related to poor hygiene**			**< 0.001**
	Bafang (n = 430)	57 (13.3)	
	Bafia (n = 421)	122 (29.0)	
	Yabassi (n = 421)	129 (30.6)	
**Have no personal opinion on transmission**			**0.006**
	Bafang (n = 430)	155 (36.1)	
	Bafia (n = 421)	119 (28.3)	
	Yabassi (n = 421)	112 (26.6)	
**Onchocerciasis perceived as “serious”**			**< 0.001**
	Bafang (n = 430)	306 (71.2)	
	Bafia (n = 421)	355 (84.3)	
	Yabassi (n = 421)	343 (81.5)	
Onchocerciasis perceived as curable*			**0.004**
	Bafang (n = 430)	399 (92.8)	
	Bafia (n = 421)	394 (93.6)	
	Yabassi (n = 421)	386 (91.7)	
Perceptions of CDTI			
**Ivermectin MDA Campaigns perceived useful and well organized**			**< 0.001**
	Bafang (n = 430)	257 (59.8)	
	Bafia (n = 421)	208 (49.4)	
	Yabassi (n = 421)	166 (39.4)	
**Community Drug Distributor perceived as "devoted and polite"**			**< 0.001**
	Bafang (n = 430)	375 (87.2)	
	Bafia (n = 421)	356 (84.6)	
	Yabassi (n = 421)	325 (77.2)	
**Ivermectin perceived effective**			0.10
	Bafang (n = 430)	307 (71.4)	
	Bafia (n = 421)	326 (77.4)	
	Yabassi (n = 421)	320 (76.0)	

* the possible answers were: yes, no, do not know

Belief that onchocerciasis is a "serious" disease was higher in Bafia and Yabassi HDs, in comparison with Bafang HD which had about 10% less of people believing that onchocerciasis is a serious disease (p<0.001, Table 2). More than 90% of the participants in the 3 HDs believed that onchocerciasis is curable (Table 2). Among those living in Bafang, only 73.4% identified ivermectin as the best treatment against onchocerciasis (Fig 1b), which was lower than the percentages registered in Bafia and Yabassi (86.8% and 79.8% respectively, p<0.001).

#### Perceptions of CDTI

The best perception on campaigns’ organization was found in Bafang HD where the proportion of people finding the campaigns “useful and well organized” was 10% higher than that in Bafia, and 20% higher than the observed proportion in Yabassi (Table 2).

The proportion of respondents who found their area’s CDDs “devoted and polite” in Bafang HD was 87.2%, which was not far from that of Bafia HD (84.6%), but about 10% higher than Yabassi HD (Table 2).

In all the three HDs, about one fourth of the participants did not see ivermectin as an effective drug. The lowest proportion of individuals perceiving ivermectin effective was found in Bafang HD (71.4%). The difference on ivermectin perceived efficacy in the three HDs was not statistically significant (Table 2).

**Fig 2:**
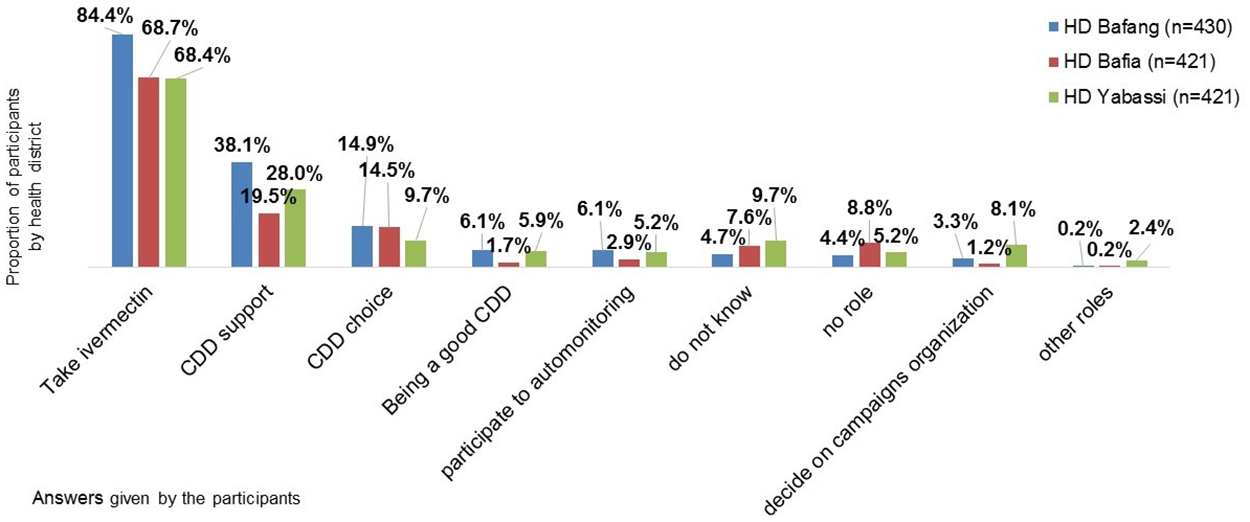
Perceived people’s role in ivermectin campaign organization system. (Multiple answers possible).

About 10% of the participants in Yabassi could not identify the community’s (population) role in the ivermectin Distribution Campaign organization system. In all the three HDs, the most perceived role of the community was to take ivermectin during the campaigns (respectively 84.4% in Bafang, 68.7% in Bafia and 68.4% in Yabassi), and those less perceived were related to the organization of activities, especially in Bafia HD (Fig 2).

### Adherence to ivermectin

#### Adherence in the 3 health districts

In all the 3 HDs the proportion of people who ever took ivermectin in their life (ivermectin users) ranged from 87 to 91% (Table 3). However, we found a low regularity among the ivermectin users: 60 to 65% took ivermectin in 2014 (the last campaign before our data collection), and 40 to 55% systematically took ivermectin each year since they started the treatment (Table 3).

**Table 3:** Ivermectin consumption flow in the HDs of Bafang, Bafia and Yabassi.

Variable	Health District	Answer given	Chi^2^ test p value
		Yes (n, %)	
**Took ivermectin at least once in the life**			0.13
	Bafang (n = 430)	383 (89.1)	
	Bafia (n = 421)	385 (91.4)	
	Yabassi (n = 421)	367 (87.2)	
**Took ivermectin during last campaign (2014 round)**			0.15
	Bafang (n = 430)	259 (60.2)	
	Bafia (n = 421)	279 (66.3)	
	Yabassi (n = 421)	274 (65.1)	
**Systematically take ivermectin during each campaign**			**<0.001**
	Bafang (n = 424)	207 (48.8)	
	Bafia (n = 393)	216 (55.0)	
	Yabassi (n = 413)	168 (40.7)	

The adherence (systematic intake) proportions were 49% (95% CI: 44–54) in Bafang, 55% (95% CI: 50–60) in Bafia, and only 41% (95% CI: 36–46) in Yabassi; p<0.001. Whereas these adherence proportions were significantly different between Bafang HD and Yabassi HD (p = 0.02), they were not significantly different between Bafang and Bafia HDs (p = 0.07).

#### Association between onchocerciasis beliefs, CDTI program perception and adherence

Not all the beliefs on onchocerciasis assessed in this study were significantly associated with regularity of ivermectin intake, whereas perceptions of CDTI programme were strongly associated with adherence, especially in the health districts of Bafang and Bafia (Table 4).

**Table 4:** Association between onchocerciasis beliefs, CDTI perception and adherence to ivermectin in Bafang, Bafia and Yabassi HDs.

	Bafang HD		Bafia HD		Yabassi HD	
	Regularly take ivermectin		Regularly take ivermectin		Regularly take ivermectin	
Beliefs on onchocerciasis	(n = 207, 48.8% of total sample)	p value	(n = 216, 55.0% of total sample)	p value	(n = 168, 40.7% of total sample)	p value*
**Perceived transmission related to poor hygiene**		0.45		0.68		0.12
*Yes n (%)*	30 (53.6)		63 (53.4)		44 (34.9)	
*No n (%)*	177 (48.1)		153 (55.6)		124 (43.2)	
**Have a personal opinion on transmission**		0.44		0.45		0.80
*No n (%)*	78 (51.3)		56 (51.9)		44 (39.6)	
*Yes n (%)*	129 (47.4)		160 (56.1)		124 (41.1)	
**Onchocerciasis perceived as "serious"**		0.81		**<0.001**		0.17
*No n (%)*	58 (47.9)		19 (31.7)		26 (33.8)	
*Yes n (%)*	149 (49.2)		197 (59.2)		142 (42.3)	
**Belief that onchocerciasis is linked to sugar**		**0.007**		**NA**		**NA**
*Yes n (%)*	53 (39.3)					
*No n (%)*	154 (53.3)					
Perceptions of CDTI programme						
**Opinion on ivermectin MDA Campaign**		**0.004**		**0.005**		0.98
*Not a positive opinion n (%)*	68 (40.2)		94 (48.0)		103 (40.7)	
*Positive opinion n (%)*	139 (54.5)		122 (61.9)		65 (40.6)	
**CDD perceived as "devoted and polite"**		**<0.001**		**<0.001**		**0.002**
*No n (%)*	11 (21.57)		18 (32.73)		25 (26.6)	
*Yes n (%)*	196 (52.55)		198 (58.58)		143 (44.83)	
**Perception of ivermectin efficacy**		**0.02**		**0.006**		0.34
*Negative n (%)*	47 (39.5)		33 (41.2)		37 (36.6)	
*Positive n (%)*	160 (52.5)		183 (58.5)		131 (42.0)	

HD, Health District; CDD, Community Drug Distributor;

*Chi^2^ test p values; NA, Not Applicable.

In Bafang, people reported a belief that onchocerciasis transmission is linked with sugar intake. Only 39.3% of them took systematically ivermectin compared to the 53.3% among those who did not share this belief (p = 0.007, Table 4). The proportions of regular ivermectin intake were higher among respondents who found onchocerciasis to be a “serious” disease, in comparison with those who didn’t perceive the disease as severe; this difference was statistically significant only in Bafia Health District (p<0.001, Table 4).

For the perceptions of CDTI programme, we found that the proportion of ivermectin systematic intake was lower among people who had a mitigated opinion (did not chose the ‘*useful and well organized*’ answer) on MDA campaigns organization, in comparison with the percentage of systematic users among those who perceived MDA campaigns as ‘*useful and well organized*’. This was namely observed in Bafang and Bafia HDs, as detailed in Table 4. In parallel, positive appreciation of CDDs was also associated with regular intake in the three HDs (p<0.005, Table 4). Perception on ivermectin efficacy also had effect on the regularity of its intake, with low proportions of regular consumers among those feeling that ivermectin is not an effective drug, in contrary to higher regular consumers among those who found ivermectin to be effective: respectively 39.5% vs 52.5% in Bafang (p = 0.02) and 41.2% vs 58.5% in Bafia (p = 0.006).

Independent factors were sought using a multivariate logistic regression model that included six variables. To obtain a “beliefs on onchocerciasis” variable, we summed the variables: perceived transmission related to hygiene, having a personal opinion on transmission, and onchocerciasis perceived as “severe”. We did the same to have a “perception on CDTI” variable, by summing opinion on MDA campaigns, CDD perception and ivermectin perceived efficacy. The other variables included in the model were the location, sex, age and the stay duration in the village.

As detailed in Table 5, we found that among all the factors identified, beliefs on onchocerciasis were the only ones not significantly related to adherence (strong misconceptions AOR = 1.15, 95% IC 0.73–1.82), whereas perceptions on CDTI programme had a strong adjusted association with adherence (best perceptions AOR = 6.82, 95% IC 3.28–14.16).

**Table 5:** Independent factors associated with adherence in three regions of Cameroon.

Variables	AOR	95% IC	p
**Perceptions on CDTI programme**			
*High*	6.82	3.29; 14.16	**<0.001**
*Medium*	4.52	2.20; 9.29	**<0.001**
*Low*	1		
**Beliefs on onchocerciasis**			
*Strong misconceptions*	1.15	0.73; 1.82	0.54
*Average misconceptions*	1.23	0.79; 1.93	0.36
*No misconceptions*	1		
**Health District**			
*Bafang*	1.42	1.06; 1.91	**0.018**
*Bafia*	1.56	1.16; 2.10	**0.003**
*Yabassi*	1		
**Sex**			
*Men*	1.33	1.04; 1.69	**0.02**
*Women*	1		
**Age**			
	1.02	1.01; 1.03	**<0.001**
**Stay duration in the village**			
	1.01	1.00; 1.02	**0.002**

AOR: adjusted odds ratio

Men were more likely to have a regular consumption of ivermectin (AOR 1.33, 95% IC 1.04–1.69). Finally, adherence also increased with age and stay duration in the village.

## Discussion

The aim of this study was to explore a possible association between people’s beliefs on onchocerciasis and perceptions of CDTI program, with their adherence to ivermectin in three Cameroonian Health Districts (HDs) that have persisting high onchocerciasis prevalence after more than 15 years of treatment.

Two main findings could be identified from this study: firstly, beliefs on onchocerciasis and their associations with adherence may differ between border regions of the country (here, 3 regions in the Centre-West area). For example, we found that in Bafang the main misconception concerning onchocerciasis transmission was a belief that it is linked with sugar consumption, while in the other HDs people mostly believed that onchocerciasis was related to hygiene conditions. The second key message is that, among all the factors related to disease and program beliefs/perceptions that were studied, perceptions of CDTI program (organization and CDD commitment) are the ones that were most determinant in adherence to ivermectin.

In this paper, we chose to study “adherence”, instead of the most commonly used “compliance” in the literature. Both terms seem to be generally used synonymously or interchangeably by many authors, as stated in Bissonnette’s analysis [23], but they are quite different. Whereas compliance has been recognized and criticized for its paternalistic connotation [23,24], adherence is preferred because of the undertone of partnership and agreement with the patient [8,23]. Furthermore, Aronson emphasises that adherence definition ‘*appropriately conjures up the tenacity that patients need to achieve in sticking to a therapeutic regiment*’ [24]. Therefore, in the frame of a long lasting community directed intervention, we thought that adherence was more appropriate than compliance. However, while citing other authors in this paper, we will keep the term “*compliance*” that they used in their publications.

The results of this study show that adherence to ivermectin was 49% (95% CI: 44–54) in Bafang, 55% (95% CI: 50–60) in Bafia, and 41% (95% CI: 36–46) in Yabassi, which are lower than the 65% minimum therapeutic coverage required by the program for onchocerciasis elimination [3]. Those results are not far from those obtained by other authors in Cameroon who found that about half of the population was compliant to ivermectin [12,22,25]. However, Senyondjo *et al*. in a recent study conducted in another HD in the west region of Cameroon, found a compliance of 71%, explainable by the fact that they observed ivermectin intake during to the last MDA campaigns prior to their study [14]. Considering this, our findings on ivermectin intake during the last MDA campaign preceding the study are comparable with the author’s results.

Literature over the past ten years (for example: Wagbatsoma *et al*. in 2004, Yirga *et al*. in 2008, Brieger *et al* in 2012, Weldegebreal *et al*. in 2014 and Afolabi *et al* in 2016) showed that huge proportions of population have misconceptions about onchocerciasis, which had important negative effect on adherence and onchocerciasis control [9,26–29]. During this period, emphasises have been laid on sensitisation and education on the disease. Consistently, in the present study it was observed that there are still non-negligible misconceptions about onchocerciasis transmission, the most common one being the link between the perception that the disease and poor hygiene are causally linked. Those misconceptions about onchocerciasis were associated with high level of education and being employed, which could be explained by the fact that people’s beliefs might be a mixture of knowledge acquired in school and the fact that most common tropical diseases are related to hygiene (for example cholera or intestinal worms). Along the same lines, Akogun *et al*. conducted a study in Nigeria that found some people “*used the information from the health education to explain their beliefs*” [30].

The surprising result which is not found in many literature is the belief that onchocerciasis is causally linked with high sugar consumption. This belief was shared by about one third of the participants in Bafang HD in addition to be the only onchocerciasis related factor significantly associated with adherence in the HD. People who had that misconception usually avoided or reduced their sugar consumption as preventive method against onchocerciasis. The harm with this misconception is that people, by reducing their sugar intake may also result in reducing their perceived risk of being ill, and consequently be less adherent to the program [16,17]. The belief was minor but not absent in the other HDs, but since this was not the objective of our study, the importance of this belief might be underestimated. Findings in Bafang HD and especially the association with adherence makes this belief that high sugar intake is one of the cause of onchocerciasis worth of interest in further qualitative and quantitative studies in order to better assess its importance, understand the explanatory mechanisms in people’s minds, and have other results on its association with adherence to ivermectin.

Perception that ivermectin MDA is ‘useful and well organized’ and that ivermectin is effective was positively associated with adherence in Bafang and Bafia HDs. These results go along with Brieger *et al*. findings in a multicountry study, where compliance was influenced by perception of ivermectin effectiveness and benefits perception in annual ivermectin treatment [9]. Perceived ivermectin MDA’s usefulness and organization could be improved with a better implication of community members in the campaigns process, and hearing their expectations towards those campaigns. In fact, in the present study organizational or leadership roles of community appeared to be not well understood by the respondents in the 3HDs: CDD choice, participation to “community auto monitoring” activities and decision on campaign organization was cited by less than 10% of them when we asked what they thought should be the community’s role. Concerning perception on ivermectin efficacy, results showed that they were influenced by participants’ opinion on its indication; most of those who gave a wrong indication of ivermectin also had a negative perception of its efficacy, probably disappointed in the expected effects after taking the drug.

Multivariable regression analysis revealed a strong association between CDTI program perception and adherence (adjusted for age, sex, location and duration of stay in the village), whereas the association between beliefs on onchocerciasis and ivermectin adherence was not significant in this study. These findings are consistent with those of recent authors who found that strong predictors of adherence were related to perception on CDTI program, especially perception of CDDs work [10,11,16,17].

A strength of our study is that we collected data from various sites in three different regions in Cameroon, with a sampling method that allowed us to reach a high geographic coverage in each district (health areas covered: 8/9 in Bafang, 13/19 in Bafia, and 7/7 in Yabassi).

Assessing adherence to ivermectin by questionnaires might be a limitation because there is the risk of respondent memory bias. However we suppose that the particularities of ivermectin tablets (small size and white colour tablets, its availability only during campaigns accompanied with sensitization and door-to-door strategy) are strong reference elements for people to recall. Consistently, Brieger *et al* in 2012 had 94% of their respondents capable of recalling up to 8 ivermectin intake in eight years [9]. Moreover Lakwo *et al*. describe a study where, the participants talking about the CDTI described a situation where they receive “*tiny tablets*” [13].

Another limitation of this study is the fact that we used a strictly quantitative approach. In fact, additional qualitative data would have been valuable to deeply explore the beliefs and perceptions expressed by the respondents: their patterns and dynamics. This was purposely done to focus on our objective, which was to weigh onchocerciasis beliefs and CDTI programme perceptions in terms of adherence to ivermectin. Qualitative data have been collected and will be analysed in the frame of another study.

Finally, also as limitation of the study we can say that since we used the term ‘filaria’ to ask about onchocerciasis during data collection, it is unclear whether people who don’t know onchocerciasis (about 41.3% of the total sample) were giving their beliefs on onchocerciasis or on other filariasis that are endemic in the same area. In 1991 and 2014 Richards *et al*., in their studies of people’s knowledges, attitudes and practices related to onchocerciasis in Guatemala, also found that most of the respondents were more familiar to the term ‘*la filaria*’ and used it for their studies [31,32] instead of the Spanish name of the disease which is ‘*oncocercosis’* [33]. However, in contrary to Guatemala, in Cameroon onchocerciasis is co-endemic with other types of filariasis, namely lymphatic filariasis and loiasis [34,35]. In 2008, the program for elimination of lymphatic filariasis started in Cameroon in all CDTI zones (including our study sites), with the association of albendazole to ivermectin during MDA. Since then, information delivered to the population emphasises on the fact that taking ivermectin and albendazole cures ‘*filaries’*. In such conditions, it can be easy for people to put both onchocerciasis and lymphatic filariasis behind the term ‘filaria’ when they describe their opinions. Nevertheless, we think that our findings remain helpful and pertinent in the present context where International Non-Governmental Development Organisations merge their efforts in order to ‘*eliminate neglected diseases in Africa*’ by enhancing MDA of ivermectin and albendazole [18].

### Conclusion

After more than 15 years of fight against onchocerciasis with the CDTI strategy, there are still frequent misconceptions about onchocerciasis transmission and ivermectin efficacy. An unexpected misconception was observed predominantly in one health district, which is the belief of a causal link between onchocerciasis and high sugar intake. The strong association between this belief and ivermectin adherence raises the need of further studies to better understand its importance and features. Perceptions of CDTI program (CDD commitment, ivermectin MDA organization and perception of ivermectin effectiveness) appeared to have a stronger association with adherence. Therefore, in addition to education/sensitisation on onchocerciasis and ivermectin use, local health authorities should also emphasise on the implementation of the CDTI in the communities, with better population ownership and ensuring of good CDD work.

## Supporting information

S1 Dataset Database for KAP survey on onchocerciasis and CDTI programme in Bafang, Bafia and Yabassi health districts. (XLSX)

S1 Checklist STROBE statement for the reporting of cross sectional studies. (DOC)
