# Supplementary material for: Adherence to ivermectin is more associated with perceptions of community directed treatment with ivermectin organization than with onchocerciasis beliefs
Source: PLoS Negl Trop Dis. 2017 Aug 14;11(8):e0005849. doi: 10.1371/journal.pntd.0005849 (PMC5570488; doi:10.1371/journal.pntd.0005849)
Supplement: S1 Checklist — (DOC) [file pntd.0005849.s002.doc]

STROBE Statement—Checklist of items that should be included in reports of ***cross-sectional studies***

|  | Item No | Details |
| --- | --- | --- |
| **Title and abstract** | 1 | **Adherence to Ivermectin: association with perceptions of Community Directed Treatment with Ivermectin programme organization and onchocerciasis beliefs in Cameroon** |
|  |  | The fight against onchocerciasis in Africa has boomed thanks to the Community Directed Treatment with Ivermectin (CDTI) program. However, in Cameroon, after more than 15 years of mass treatment, onchocerciasis prevalence is still above the non-transmission threshold. This study aimed to explore a possible association between people’s beliefs/perceptions of onchocerciasis and of CDTI program, and their adherence to ivermectin in three regions of Cameroon. A cross sectional survey was carried out in three health districts with persistent high onchocerciasis prevalence. Participants were randomly selected in 30 clusters per district. Adherence to ivermectin was comparable between Bafang and Bafia (55.0% and 48.8%, respectively, p>0.05) and lower in Yabassi (40.7%). Among all factors related to program perceptions and disease representations that were studied, perceptions of the program are the ones that were most determinant in adherence to ivermectin. People who had a “not positive” opinion of ivermectin distribution campaigns were less compliant than those who had a positive opinion about the campaigns (40% vs 55% in Bafang, and 48% vs 62% in Bafia, p<0.01), as well as those who had a negative appreciation of community drug distributors’ commitment (22% vs 53% in Bafang, 33% vs 59% in Bafia, 27% vs 47% in Yabassi; p<0.01). The most common misconception about onchocerciasis transmission was the lack of hygiene, especially in Bafia and Yabassi. In Bafang, high proportions of people believed that onchocerciasis was due to high consumption of sugar (31% vs less than 5% in Bafia and Yabassi, p<0.001). In conclusion, there are still frequent misconceptions about onchocerciasis transmission in Cameroon. Perceptions of ivermectin distribution campaigns are more strongly associated to adherence. In addition to education/sensitisation on onchocerciasis during the implementation of the CDTI program, local health authorities should strive to better involve communities and more encourage community distributors’ work. |
| Introduction | | |
| Background/rationale | 2 | Onchocerciasis control in Africa has boomed with the ‘Community Directed Treatment with Ivermectin’ program. Successes of this program over many African countries prompted the international community to set it as main strategy for elimination of onchocerciasis and lymphatic filariasis. In Cameroon, onchocerciasis prevalence in some areas is still higher than what was predicted, despite more than 15 years of mass treatment. Therefore, in the frame of enlargement of the treatment zones, there is a need for enriching local literature on modifiable factors that influence adherence to ivermectin. |
| Objectives | 3 | Our core objectives were to:   - Assess possible association between systematic ivermectin intake and people’s beliefs of the disease in three neighbouring regions of the country. - Assess possible association between systematic ivermectin intake and people’s perceptions of the program in three neighbouring regions of the country. - Determine most strongly associated factors of adherence to ivermectin in order to inform policy.   In fact, as hypothesis, we assumed that if despite 15 years of mass drug administration onchocerciasis prevalence is still above the expected values, this may be due to poor adherence to ivermectin (people are not/not well taking the medication). This hypothesized poor adherence could be explained firstly by the persistence of misconceptions about the disease. The second possibility was that there might be some unmet peoples’ expectations towards the management of the disease. |
| Methods | | |
| Study design | 4 | We conducted a cross sectional cluster study, with 30 clusters in each health district corresponding to 30 different communities. These communities were randomly selected using a proportional-to-population size method based on sizes from the 2014 CDD census in the targeted HDs. Data collection was done using a pre tested questionnaire administered to participants by trained research assistants. |
| Setting | 5 | Data were collected between April and June 2015 in three border regions in the Centre-West area of Cameroon. In the West Region we choose the Health District of Bafang, in the Centre Region the Health District of Bafia, and in the Littoral Region, the Health District of Yabassi. Those Health districts are part of the Community Directed Treatment with Ivermectin program in Cameroon. |
| Participants | 6 | To obtain the estimated number of individuals in each Health District, we selected 30 clusters, corresponding to 30 different communities. These communities were randomly selected using a proportional-to-population size method based on sizes from the 2014 program census in the targeted HDs.  Since it is quite difficult to get an accurate list of households, especially in the rural areas of Cameroon, the households in each community were randomly selected by the research assistant who took the direction indicated by the bottle neck after turning a bottle on the ground, from a central point within the community (the health centre of the village or the chief’s house) and taking all households located in his right side until reaching the required number of households (421 households to have 421 respondents). In the event that he found an endpoint, he returned to the main point of the village and repeated the bottle operation. In each household, the respondent was selected among the present eligible members by tossing a coin, after the approval of the head of household. In case the head of household rejected the toss, he/she was given the opportunity to select the respondent of his choice.  All individuals found in the households were eligible for the interview except: visitors, people aged less than 15 years and those who couldn’t understand nor answer in French. |
| Variables | 7 | The outcome (dependant variable) in this study was adherence to ivermectin, also called systematic intake of ivermectin. This was obtained by asking to the participants if they systematically took the ivermectin distributed during the mass drug administration campaigns since they started to take it (yes/no). |
| Data sources/ measurement | 8* | Variables of interest included sociodemographic factors, disease related factors, and program related factors.   - Socio demographic factors included, for each Health District were: age, sex, educational level, main occupation, and duration of stay in the village. - Disease related factors were assessed by asking people’s beliefs on onchocerciasis transmission (open ended multiple choice question) and onchocerciasis severity (yes/no/don’t know question). - Program perceptions were assessed by seeking respondent’s perception of ivermectin distribution campaigns’ usefulness and good organization, their perception of community drug distributors devotion and politeness (open ended multiple choice question), and their opinion on ivermectin efficacy (yes/no/don’t know)   For the purpose of multivariate analysis, we built ‘perceptions on CDTI’ and ‘beliefs on onchocerciasis’ variables.   - ‘Perceptions on CDTI’ was obtained by adding the 3 above named program perception variables. Then we grouped the results as follows: 0 = low perceptions 1-2 = medium perceptions and 3 = high perceptions. - “Beliefs on onchocerciasis” was obtained by adding the two main misconceptions on onchocerciasis transmission (poor hygiene and ‘I have no personal opinion’) and the variable on opinion on onchocerciasis severity. The results were grouped as follows: 0 = no misconceptions 1-2 = average misconceptions and 3 = strong misconceptions. |
| Bias | 9 | Potential sources of bias:   - Selection bias was controlled firstly by the choice of different clusters in different communities all over the Health Districts. Secondly, choose only one respondent per household, and finally we randomly selected the respondent in each household (instead of systematically take the household head which most commonly corresponds to adult men). - Memory bias can be discussed here, but we think that the mobilization that surrounds the campaigns, and the fact that drugs are received door-to-door make the event memorable. Moreover, some authors talked about the size of ivermectin (very small tablets) as another mind map to recall ivermectin intake [1]. Other authors confronted their participants’ memory to the local health records and had up to 94% of concordance concerning the recall of ivermectin consumption over 8 years [2]. |
| Study size | 10 | Sample size computation in each Health District (HD) was based on the hypothesis that at least 50% (non-informative prior prevalence) of respondents will have a positive perception on onchocerciasis and Community Directed Treatment with Ivermectin program, using a 10% margin error, a design effect of 4 for correcting for a high intra clusters correlation, and assuming a non-respondent loss rate of 10%. Under such assumptions, the minimal size for each HD was 421 subjects. |
| Quantitative variables | 11 | Our two quantitative data, age and duration of stay in the village, were kept in their original form.   - For descriptive results we used the median and IQR (not normal distribution). - For measure of association and multivariate analysis we used the logistic regression. |
| Statistical methods | 12 | We used the Chi square test to compare the difference between the proportions, and multivariate logistic regression to obtain adjusted odds ratios and assess eventual confounding factors.  All the analysis were performed using the STATA v. 13 software. |
|  |  | To avoid non responses bias, we assumed a non-respondent loss rate of 10% in the sample size calculation. Missing data were not considered during data analysis, and during the presentation of the results we always precise the sample size in brackets. |
| Results | | |
| Participants | 13* | Across the 3 Health Districts 1,378 households were identified, amongst which 89 (6.5%) were excluded because of participation refusal. The main reasons of refusal were: busy schedule (going to farm, cooking), tiredness (coming from farm/work), or lack of interest on the study. Only 17 households (1.2%) were excluded because of local language barriers. Finally, we visited 1,272 households (representing 92.3% of the total eligible households). One member per household was interviewed, making a final sample size of 1,272 individuals: 430 in Bafang, 421 in Bafia, and 421 in Yabassi. |
|  |  | Flow diagram of participants in the study (next page)  Opened households: *N=1378*  **Final sample: n=1272**  Head of household refused to participate: *n_1_= 89 (6.46%)*  Head of household agreed to participate (n_2_=1289)  Language barrier (local language): *n_3_=17*  Legend:  : Included : Excluded |
| Descriptive data | 14* | Characteristics of study participants (eg demographic, clinical, social)   |  |  | **Health district** | | | | --- | --- | --- | --- | --- | |  |  | **Bafang** | **Bafia** | **Yabassi** | |  | **(n=430)** | **(n=421)** | **(n=421)** | | **Age, years** | |  |  |  | |  | *Median (IQR)* | 35 (24-50) | 40 (27-52) | 37 (27-52) | |  | *Range* | 15-82 | 15-86 | 15-90 | | **Sex, n (%)** | |  |  |  | |  | *Female* | 247 (57.4) | 197 (46.8) | 205 (48.7) | | **Level of instruction, n (%)** | |  |  |  | |  | *No formal* | 28 (6.5) | 35 (8.3) | 32 (7.6) | |  | *Primary* | 92 (21.4) | 164 (39.0) | 131 (31.1) | |  | *Secondary 1* | 137 (31.9) | 122 (29.0) | 125 (29.7) | |  | *Secondary 2 and above* | 173 (40.23) | 100 (23.75) | 133 (31.6) | | **Principal occupation, n (%)** | |  |  |  | |  | *No occupation* | 207 (48.1) | 161 (38.2) | 182 (43.2) | |  | *Farmer/trader* | 171 (39.8) | 216 (51.3) | 180 (42.8) | |  | *Employed* | 52 (12.1) | 44 (10.5) | 59 (14.0) | | **Length of stay in the village, years** | | |  |  | |  | *Median (IQR)* | 14 (6-26) | 20 (9-39) | 13 (4-31) | |  | *Range* | 0-82 | 0-80 | 0-80 | |
|  |  | Number of participants with missing data for each variable of interest   - **Age**   Number of participants: 1,272  Missing data: 1 (0.08%)   - **Sex**   Number of participants: 1,272  Missing data: 0 (0%)   - **Level of instruction**   Number of participants: 1,272  Missing data: 0 (0%)   - **Principal occupation**   Number of participants: 1,272  Missing data: 0 (0%)   - **Length of stay in the village**   Number of participants: 1,272  Missing data: 0 (0%)   - **Beliefs about onchocerciasis transmission**:   Number of participants: 1,272  Missing data: 11 (0.86%)   - **Opinion on onchocerciasis severity**   Number of participants: 1,272  Missing data: 0 (0%)   - **Perception of ivermectin distribution campaigns’ usefulness and good organization**   Number of participants: 1,272  Missing data: 1 (0.08%)   - **Perception of community drug distributors devotion and politeness**   Number of participants: 1,272  Missing data: 0 (0%)   - **Opinion on ivermectin efficacy**   Number of participants: 1,272  Missing data: 0 (0%) |
| Outcome data | 15* | Outcome event: **Systematic intake of ivermectin**  Number of participants: 1272  Missing data: 42 (3.3%)  Systematic intake Bafang: 48.8% (95% CI: 44.1-53.6)  Systematic intake Bafia: 55.0% (95% CI: 50.0-59.8)  Systematic intake Yabassi: 40.7% (95% CI: 36.0-45.5) |
| Main results | 16 | Our main objective was to assess possible association between modifiable factors, namely beliefs/perceptions on onchocerciasis/CDTI program organization.  As shown in the table below, we found that adherence to ivermectin increased with the perception of CDTI program organization. However, there was no significant association between beliefs on onchocerciasis and adherence to ivermectin.   | Variables | | COR | 95% IC | | p | | --- | --- | --- | --- | --- | --- | | **Perceptions on CDTI programme** | |  |  |  |  | |  | *High* | 6.87 | 3.41 | 13.85 | **<0.001** | |  | *Medium* | 4.40 | 2.20 | 8.79 | **<0.001** | |  | *Low* | 1 |  |  |  | | **Beliefs on onchocerciasis** | |  |  |  |  | |  | *Strong misconceptions* | 1.41 | 0.916 | 2.165 | 0.12 | |  | *Average misconceptions* | 1.36 | 0.887 | 2.073 | 0.16 | |  | *No misconceptions* | 1 |  |  |  | | *COR: crude (unadjusted) odds ratio* | |  |  |  |  |   We adjusted in a multivariable logistic regression model, where we included the location (health district), age, sex, and duration of stay in the village. Those factors, mostly non modifiable, were included in the model because of their mentioned association with adherence in the literature. We also added the health districts to assess possible differences of adherence within neighbouring regions in the same country. As detailed in the table below, the strength of the association between perception of CTDI program organization and adherence to ivermectin remained unchanged. All the other factors included in the model were significantly associated to adhesion, except beliefs on onchocerciasis.   | Variables | | AOR | 95% IC | | p | | --- | --- | --- | --- | --- | --- | | **Perceptions on CDTI programme** | |  |  |  |  | |  | *High* | 6.82 | 3.29 ; | 14.16 | **<0.001** | |  | *Medium* | 4.52 | 2.20 ; | 9.29 | **<0.001** | |  | *Low* | 1 |  |  |  | | **Beliefs on onchocerciasis** | |  |  |  |  | |  | *Strong misconceptions* | 1.15 | 0.73 ; | 1.82 | 0.54 | |  | *Average misconceptions* | 1.23 | 0.79 ; | 1.93 | 0.36 | |  | *No misconceptions* | 1 |  |  |  | | **Health District** | |  |  |  |  | |  | *Bafang* | 1.42 | 1.06 ; | 1.91 | **0.018** | |  | *Bafia* | 1.56 | 1.16 ; | 2.10 | **0.003** | |  | *Yabassi* | 1 |  |  |  | | **Sex** | |  |  |  |  | |  | *Men* | 1.33 | 1.04 ; | 1.69 | **0.02** | |  | *Women* | 1 |  |  |  | | **Age** | |  |  |  |  | |  |  | 1.02 | 1.01 ; | 1.03 | **<0.001** | | **Stay duration in the village** | |  |  |  |  | |  |  | 1.01 | 1.00 ; | 1.02 | **0.002** | |  | *AOR: adjusted odds ratio* |  |  |  |  | |
| Other analyses | 17 | Report other analyses done—eg analyses of subgroups and interactions, and sensitivity analyses  We evaluated the distribution of beliefs on onchocerciasis and perceptions of CDTI program organization within the health districts, and found that to the question about identification of onchocerciasis (filaria) causes, only 15.6% cited black fly bite in Bafang, as against 29.7% and 31.4 % in Bafia and Yabassi respectively, p<0.001. Higher proportion of people believing that filaria was caused by a high sugar consumption or high blood sugar levels were also found in Bafia (31.4%, versus 1.0% and 3.1% in Bafia and Yabassi respectively, p<0.001). The most common misconception about onchocerciasis transmission was related to poor hygiene conditions, namely in Bafia and Yabassi where respectively 29.0% and 30.6% shared that misconception as against 13.3% in Bafang (p<0.001). Belief that onchocerciasis is a "serious" disease was higher in Bafia and Yabassi HDs, in comparison with Bafang HD which had about 10% less of people believing that onchocerciasis is a serious disease (p<0.001).  Concerning program perceptions, the best perception on campaigns’ organization was found in Bafang HD where the proportion of people finding the campaigns “useful and well organized” was 10% higher than that in Bafia, and 20% higher than the observed proportion in Yabassi.  The proportion of respondents who found their area’s CDDs “devoted and polite” in Bafang HD was 87.2%, which was not far from that of Bafia HD (84.6%), but about 10% higher than Yabassi HD (Table 2). In all the three HDs, about one fourth of the participants did not see ivermectin as an effective drug. The lowest proportion of individuals perceiving ivermectin effective was found in Bafang HD (71.4%). The difference on ivermectin perceived efficacy in the three HDs was not statistically significant (p<0.001). About 10% of the participants in Yabassi could not identify the community’s (population) role in the ivermectin Distribution Campaign organization system. In all the three HDs, the most perceived role of the community was to take ivermectin during the campaigns (respectively 84.4% in Bafang, 68.7% in Bafia and 68.4% in Yabassi), and those less perceived were related to the organization of activities, especially in Bafia HD.  The analysis of association between onchocerciasis beliefs, CDTI program perception and adherence within the health districts revealed that not all the beliefs on onchocerciasis assessed in this study were significantly associated with regularity of ivermectin intake, whereas perceptions of CDTI programme were strongly associated with adherence, especially in the health districts of Bafang and Bafia. In Bafang, people reported a belief that onchocerciasis transmission is linked with sugar intake. Only 39.3% of them took systematically ivermectin compared to the 53.3% among those who did not share this belief (p=0.007). The proportions of regular ivermectin intake were higher among respondents who found onchocerciasis to be a “serious” disease, in comparison with those who didn’t perceive the disease as severe; this difference was statistically significant only in Bafia Health District (p<0.001).  Furthermore, the proportion of ivermectin systematic intake was lower among people who had a mitigated opinion on ivermectin distribution campaigns organization, in comparison with the percentage of systematic users among those who perceived MDA campaigns as ‘*useful and well organized*’. This was namely observed in Bafang and Bafia HDs, as detailed in table 4. In parallel, positive appreciation of community drug distributors was also associated with regular intake in the three HDs (p<0.005). Perception on ivermectin efficacy also had effect on the regularity of its intake, with low proportions of regular consumers among those feeling that ivermectin is not an effective drug, in contrary to higher regular consumers among those who found ivermectin to be effective: respectively 39.5% vs 52.5% in Bafang (p=0.02) and 41.2% vs 58.5% in Bafia (p=0.006). |
| Discussion | | |
| Key results | 18 | The results of this study show that adherence to ivermectin was 48.8% (95% CI: 44.1-53.6) in Bafang, 55% (95% CI: 50.0-59.8) in Bafia, and 40.7% (95% CI: 36.0-45.5) in Yabassi, which are not far from those obtained by other authors in Cameroon who found that about half of the population was compliant to ivermectin [3-5]. Multivariable regression analysis revealed a strong association between CDTI program perception and adherence (adjusted for age, sex, location and duration of stay in the village), whereas the association between beliefs on onchocerciasis and ivermectin adherence was not significant in this study. These findings are consistent with those of recent authors who found that strong predictors of adherence were related to perception on CDTI program, especially perception of CDDs work [6-9]. |
| Limitations | 19 | Assessing adherence to ivermectin by questionnaires might be a limitation because there is the risk of respondent memory bias. However we suppose that the particularities of Mectizan® tablets (small size and white colour tablets, its availability only during campaigns accompanied with sensitization and door-to-door strategy) are strong reference elements for people to recall. Consistently, Brieger *et al* in 2012 had 94% of their respondents capable of recalling up to 8 Mectizan® intake in eight years [2]. Moreover Lakwo *et al*. describe a study where, the participants talking about the CDTI described a situation where they receive “*tiny tablets*” [1].  Another limitation of this study is the fact that we used a strictly quantitative approach. In fact, additional qualitative data would have been valuable to deeply explore the beliefs and perceptions expressed by the respondents: their patterns and dynamics. This was purposely done to focus on our objective, which was to weigh onchocerciasis beliefs and CDTI programme perceptions in terms of adherence to ivermectin. Qualitative data have been collected and will be analysed in the frame of another study. |
| Interpretation | 20 | One caution that can be taken in account in the interpretation of our results resides on the fact that since we used the term ‘filaria’ to ask about onchocerciasis during data collection, it is unclear whether people who don’t know onchocerciasis (about 41.3% of the total sample) were giving their beliefs on onchocerciasis or on other filariasis that are endemic in the same area. In 1991 and 2014 Richards *et al.*, in their studies of people’s knowledges, attitudes and practices related to onchocerciasis in Guatemala, also found that most of the respondents were more familiar to the term ‘*la filaria*’ and used it for their studies [10,11] instead of the Spanish name of the disease which is ‘*oncocercosis’* [12]. However, in contrary to Guatemala, in Cameroon onchocerciasis is co-endemic with other types of filariasis, namely lymphatic filariasis and loiasis [13,14]. In 2008, the program for elimination of lymphatic filariasis started in Cameroon in all CDTI zones (including our study sites), with adjunction of albendazole to ivermectin during MDA. Since then, information delivered to the population emphasises on the fact that taking ivermectin and albendazole cures ‘*filaries’*. In such conditions, it can be easy for people to put both onchocerciasis and lymphatic filariasis behind the term ‘filaria’ when they describe their opinions. Nevertheless, we think that our findings remain helpful and pertinent in the present context where International Non-Governmental Development Organisations merge their efforts in order to ‘*eliminate neglected diseases in Africa*’ by enhancing MDA of ivermectin and albendazole [15]. |
| Generalisability | 21 | Discuss the generalisability (external validity) of the study results.  Literature over the past ten years showed huge misconceptions about onchocerciasis, which had important negative effect on adherence to ivermectin and thus, on onchocerciasis control. Since then and up to now, emphasises is laid on sensitisation and education about the disease, especially during campaigns. The present study comes in reinforcement of recent studies who show that the perception on how the program is implemented has a great importance on ivermectin adherence. The fact that we have similar results in different contexts makes us to assume the generalisability of these result to sub-Saharan context. |
| Other information | | |
| Funding | 22 | The study was funded by the Belgian Académie de Recherche et d’Enseignement Supérieur (ARES-CCD: www.ares-ac.be) through the 2013 Research Project for development (PRD) in Cameroon, named: PRD2013Cameroun-Souopgui- Strenghtening the Onchocerciasis Elimination Program in Cameroon. More information on: <http://www.ares-ac.be/fr/cooperation-au-developpement/pays-projets/projets-dans-le-monde/item/54-prd-strengthening-the-onchocerciasis-elimination-program-in-cameroon>  The funders had no role in study design, data collection and analysis, decision to publish, or preparation of the manuscript. |

**REFERENCES**

1. Lakwo TL, Gasarasi DB. Non-adherence to community directed treatment with ivermectin for onchocerciasis control in Rungwe district, Southwest Tanzania. East Afr Med J. 2006;83(6):326–32.
2. Brieger WR, Okeibunor JC, Abiose AO, Ndyomugyenyi R, Wanji S, Elhassan E, et al. Characteristics of persons who complied with and failed to comply with annual ivermectin treatment: Who complies with annual ivermectin treatment? Trop Med Int Health. 2012 Jul;17(7):920–30.
3. Brieger WR, Okeibunor JC, Abiose AO, Wanji S, Elhassan E, Ndyomugyenyi R, et al. Compliance with eight years of annual ivermectin treatment of onchocerciasis in Cameroon and Nigeria. Parasit Vectors. 2011;4:152.
4. Kamga G-R, Dissak-Delon FN, Nana-Djeunga HC, Biholong BD, Mbigha-Ghogomu S, Souopgui J, et al. Still mesoendemic onchocerciasis in two Cameroonian community-directed treatment with ivermectin projects despite more than 15 years of mass treatment. Parasit Vectors. 2016;9:581.
5. Epee E, Afetane TG, Aboutou R, Assumpta LB. Lésions oculaires de l’onchocercose et observance au Traitement à L’Ivermectine Sous Directives Communautaires (TIDC). Health Sci Dis [Internet]. 2014 Apr 25;15(2). Available from: <http://www.hsd-fmsb.org/index.php/hsd/article/view/338>
6. Endale A, Erko B, Weldegebreal F, Legesse M. Predictors of compliance with community-directed treatment with ivermectin for onchocerciasis control in Kabo area, southwestern Ethiopia. Parasit Vectors [Internet]. 2015 Feb 15 [cited 2016 May 20];8. Available from: <http://www.ncbi.nlm.nih.gov/pmc/articles/PMC4335633/>
7. Amazigo U, Okeibunor J, Matovu V, Zouré H, Bump J, Seketeli A. Performance of predictors: Evaluating sustainability in community-directed treatment projects of the African programme for onchocerciasis control. Soc Sci Med. 2007 May;64(10):2070–82.
8. Yirga D, Deribe K, Woldemichael K, Wondafrash M, Kassahun W. Factors associated with compliance with community directed treatment with ivermectin for onchocerciasis control in Southwestern Ethiopia. Parasit Vectors. 2010 Jun 2;3:48.
9. Nuwaha F, Okware J, Ndyomugyenyi R. Predictors of compliance with community-directed ivermectin treatment in Uganda: quantitative results. Trop Med Int Health. 2005 Jul 1;10(7):659–67.
10. Richards F, Klein RE, Gonzales-Peralta C, Zea Flores R, Zea Flores G, Castro Ramirez J. Knowledge, Attitudes and Perceptions (KAP) of onchocerciasis: A survey among residents in an endemic area in Guatemala targeted for mass chemotherapy with ivermectin. Soc Sci Med. 1991 Jan 1;32(11):1275–81.
11. Richards FOJ, Klein RE, León O de, Mendizábal-Cabrera R, Morales AL, Cama V, et al. A Knowledge, Attitudes and Practices Survey Conducted Three Years after Halting Ivermectin Mass Treatment for Onchocerciasis in Guatemala. PLOS Negl Trop Dis. 2016 Jun 24;10(6):e0004777.
12. Mitchell C. OPS OMS | Oncocercosis [Internet]. Pan American Health Organization / World Health Organization. [cited 2016 Oct 13]. Available from: <http://www.paho.org/hq/index.php?option=com_content&view=article&id=9473%3A2014-onchocerciasis&catid=6648%3Afact-sheets&Itemid=40721&lang=es>
13. Nana-Djeunga HC, Tchatchueng-Mbougua JB, Bopda J, Mbickmen-Tchana S, Elong-Kana N, Nnomzo’o E, et al. Mapping of Bancroftian Filariasis in Cameroon: Prospects for Elimination. PLoS Negl Trop Dis [Internet]. 2015 Sep 9 [cited 2016 Oct 8];9(9). Available from: <http://www.ncbi.nlm.nih.gov/pmc/articles/PMC4564182/>
14. Zouré HGM, Wanji S, Noma M, Amazigo UV, Diggle PJ, Tekle AH, et al. The Geographic Distribution of Loa loa in Africa: Results of Large-Scale Implementation of the Rapid Assessment Procedure for Loiasis (RAPLOA). PLOS Negl Trop Dis. 2011 Jun 28;5(6):e1210.
15. Programme Africain de Lutte contre l’Onchocercose (OMS/APOC). Programme pour l’élimination des maladies négligées en Afrique (PENDA). Plan d’action stratégique et budget indicatif 2016-2025 [Internet]. WHO. 2013 [cited 2016 Sep 30]. Available from: <http://www.who.int/entity/apoc/fr_strategic_plan_2013_ok.pdf?ua=1>
